# To What Extent Are the Terminal Stages of Sepsis, Septic Shock, Systemic Inflammatory Response Syndrome, and Multiple Organ Dysfunction Syndrome Actually Driven by a Prion/Amyloid Form of Fibrin?

**DOI:** 10.1055/s-0037-1604108

**Published:** 2017-08-04

**Authors:** Douglas B. Kell, Etheresia Pretorius

**Affiliations:** 1 School of Chemistry, The University of Manchester, Manchester, United Kingdom; 2 Manchester Institute of Biotechnology, The University of Manchester, Manchester, United Kingdom; 3 Centre for Synthetic Biology of Fine and Speciality Chemicals, The University of Manchester, Manchester, United Kingdom; 4 Department of Physiological Sciences, Stellenbosch University, Matieland, South Africa

**Keywords:** sepsis, SIRS, dormant bacteria, septic shock, MODS

## Abstract

A well-established development of increasing disease severity leads from sepsis through systemic inflammatory response syndrome, septic shock, multiple organ dysfunction syndrome, and cellular and organismal death. Less commonly discussed are the equally well-established coagulopathies that accompany this. We argue that a lipopolysaccharide-initiated (often disseminated intravascular) coagulation is accompanied by a proteolysis of fibrinogen such that formed fibrin is both inflammatory and resistant to fibrinolysis. In particular, we argue that the form of fibrin generated is amyloid in nature because much of its normal α-helical content is transformed to β-sheets, as occurs with other proteins in established amyloidogenic and prion diseases. We hypothesize that these processes of amyloidogenic clotting and the attendant coagulopathies play a role in the passage along the aforementioned pathways to organismal death, and that their inhibition would be of significant therapeutic value, a claim for which there is considerable emerging evidence.

Sepsis is a disease with high mortality. 1 2 3 4 5 6 7 However, the original notion of sepsis as the invasion of blood and tissues by pathogenic microorganisms has long come to be replaced, in the antibiotic era, by the recognition that in many cases, the main causes of death arise not so much from the replication of the pathogen per se but from the host's “innate immune” *response* to the pathogen. 8 9 10 11 In particular, microbial replication is not even necessary (and most bacteria in nature are dormant 12 13 14 15 16 ), as this response is driven by very potent 17 inflammation-inducing agents such as the lipopolysaccharides (LPSs) of gram-negative bacteria 18 and equivalent cell wall materials such as lipoteichoic acids from gram-positive bacteria. 19 20 21 22 To this end, such release may even be worsened (i.e., the Jarisch–Herxheimer reaction 23 24 25 26 ) by antibiotic therapy. 27 28 29 30 In unfavorable cases, this leads to an established cascade ( Fig. 1 ) 31 in which the innate immune response, involving proinflammatory cytokines such as interleukins 6, 8, and 1β, monocyte chemoattractant protein-1, and tissue necrosis factor α, 32 becomes a “cytokine storm” 33 34 35 36 37 leading to a “systemic inflammatory response syndrome” (SIRS), 38 39 40 41 42 43 septic shock, 4 multiple organ failure 44 (MOF, also known as multiple organ dysfunction syndrome, MODS 45 46 ), and finally organismal death. All of the above is well known and may be taken as a noncontroversial background. Nevertheless, it is still unclear whether apoptotic 47 and necrotic 48 cell death is minimal 49 or significant. Despite this knowledge, “the recent inability of activated protein C to show an outcome benefit in a randomized controlled multicenter trial 2 and the subsequent withdrawal of the product from commercial use add to the growing stockpile of failed therapeutics for sepsis.” 50 (This last failure was probably due to an excessively anticoagulant activity.)

**Fig. 1:**
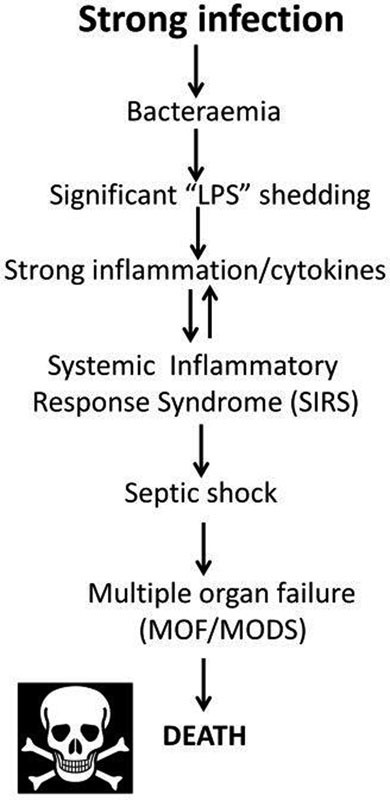
A standard cascade illustrating the progression of infection through sepsis, systemic inflammatory response syndrome, and death.

Most recently 51 (but see also Churpek et al 52 ), definitions of sepsis have come to be based on organ function and the Sequential (Sepsis-Related) Organ Failure Assessment (SOFA) Scores. 53 These latter take into account the multisystem nature of sepsis and include respiratory, hemostatic (but only based on platelet counts), hepatic, cardiovascular, renal, and central nervous system measurements. A SOFA score of 2 or greater typically means at least a 10% mortality rate. Specifically, sepsis is defined as a life-threatening organ dysfunction caused by a dysregulated host response to infection. Septic shock is defined as a subset of sepsis in which underlying circulatory and cellular metabolism abnormalities are profound enough to increase mortality substantially.

Table 1 (based on Vincent et al 53 ) shows the potential values that contribute to the SOFA score.

**Table 1:** Potential values that contribute to the SOFA score a

SOFA score	1	2	3	4
*Respiration* PaO _2_ /FiO _2_ (mm Hg)	<400	<300	<200 (with respiratory support)	<100 (with respiratory support)
*Coagulation* 10 ^−3^ /platelets/mm	<150	<100	<50	<50
*Liver* Bilirubin mg/dL (μM)	1.2–1.9 (20–32)	2–5.9 (33–101)	6–11.9 (102–204)	>12 (>204)
*Cardiovascular* Hypotension	MA *p* < 70 mm Hg	Dopamine ≤ 5 b or dobutamine (any dose)	Dopamine > 5 or epinephrine ≤0.1 or norepinephrine ≤ 0.1	Dopamine > 15 or epinephrine > 0.1 or norepinephrine > 0.1
*CNS* Glasgow Coma Score	13–14	10–12	6–9	<6
*Renal* Creatinine, mg/dL (μM) or urine output	1.2–1.9 (110–170)	2–3.4 (171–299)	3.5–4.9 (300–440) Or <500 mL/d	>5 (>440) or <200 mL/d

Abbreviations: CNS, central nervous system; SOFA, Sequential (Sepsis-Related) Organ Failure Assessment.

a Based on Vincent et al 53 and shows the potential values that contribute to the SOFA score.

b Catecholamine and adrenergic agents administered for at least 1 hour; doses in μg/kg/min.

Absent from Fig. 1 , and from the usual commentaries of this type, is any significant role of coagulopathies, although these too are a well-established accompaniment of SIRS/sepsis, 54 55 56 57 58 59 60 61 62 63 64 65 66 67 68 69 and they will be our focus here. They form part of an emerging systems biology analysis, 16 18 70 71 72 73 74 75 76 77 78 79 80 in which iron dysregulation and an initially minor infection (e.g., in rheumatoid arthritis 81 82 ) are seen to underpin the etiology of many chronic inflammatory diseases normally considered (as once were gastric ulcers 83 ) to lack a microbial component.

Here we develop these ideas for those conditions that are recognized as involving a genuine initial microbial invasion, together with sepsis and inflammation driven (in particular) by the cell wall components of bacteria (although we note that the same kinds of arguments apply to viruses 84 and to other infections).

## Normal Blood Coagulation and Coagulopathies

**Fig. 2:**
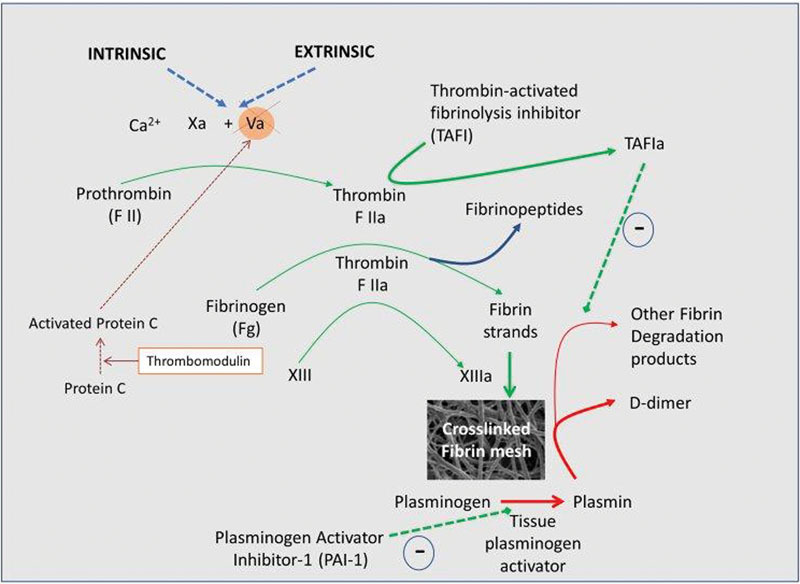
The classical coagulation pathways, where assembly of fibrin fibers proceeds in a stepwise fashion (redrawn from Kell and Pretorius 74 under an open access CC-BY license).

**Fig. 3:**
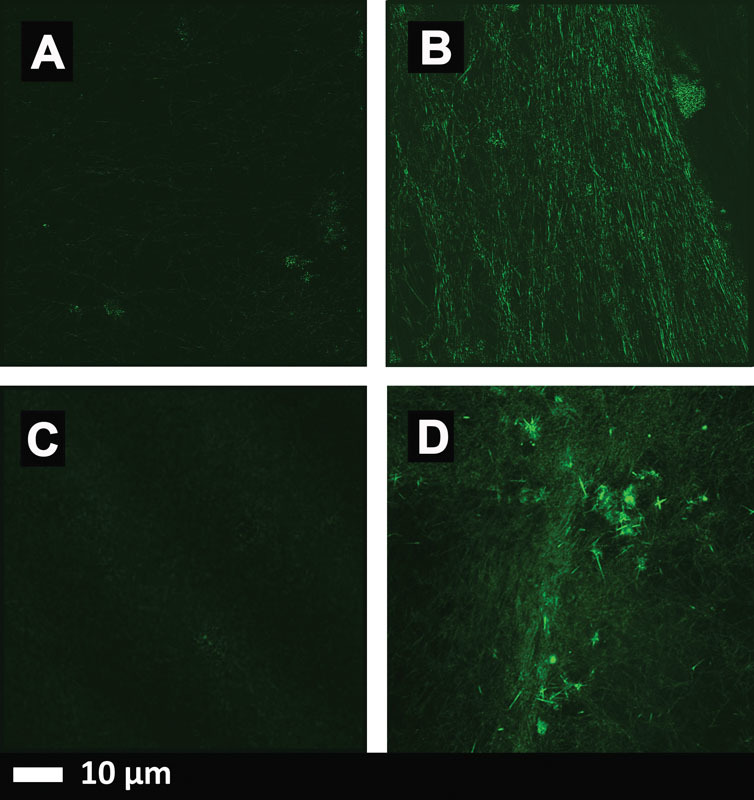
The results of thrombin-mediated blood clotting. **(A–C)** Micrographs taken with a Zeiss LSM 800 superresolution Airyscan confocal microscopy using the α Plan-Apochromat 63x/1.46 Oil DIC M27 Elyra objective. **(D)** Micrograph taken with a Zeiss LSM 510 META confocal microscope with a Plan-Apochromat 63x/1.4 Oil DIC objective. ( **A** ) Healthy platelet poor plasma (PPP) with added thioflavin T (ThT) (5-μM exposure concentration) and thrombin. ( **B** ) The same PPP, with added lipopolysaccharide (LPS) (0.2 ng/L exposure concentration), followed by addition ThT and thrombin. ( **C** ) The same PPP, with added LPS followed by LPS-binding protein (2 ng/L final exposure concentration) followed by addition ThT and thrombin. ( **D** ) PPP, with added LPS (0.2 ng/L exposure concentration), followed by addition ThT and thrombin.

Historically, there are two main pathways of activation described that lead “normal” blood coagulation to form a clot, as occurs, for example, in response to vessel wall damage or exposure of blood to negatively charged surfaces. They have been expertly reviewed many times 85 86 87 88 89 90 (e.g., are known in the older literature, respectively, as “extrinsic” and “intrinsic” pathways). Fig. 2 shows a basic model of coagulation (redrawn from Kell and Pretorius 74 under a CC-BY license); typically, assembly of fibrin fibers proceeds in a stepwise fashion. In short, after damage to a blood vessel, collagen is exposed and factor (F) VII interacts with tissue factor (TF), forming a complex called TF-FVIIa. FXa and its cofactor Va form the prothrombinase complex and activate thrombin through prothrombin. Finally, the terminal stages of the coagulation pathway happens, where a cross-linked fibrin polymer is formed as a result of fibrinogen (typically present in plasma at 2–4 g/L) conversion to fibrin and cross-linking due to the activation of FXIII, a transglutaminase. Thrombin activates FXIII into FXIIIa, which, in turn, acts on soluble and insoluble fibrin to polymerize it into insoluble cross-linked fibrin clot. This fibrin clot, when viewed under a scanning electron microscope, consists of individually visible fibrin fibers, discussed in the next paragraphs (see Fig. 3A for a representative healthy clot structure created when thrombin is added to plasma. 74 91 92 93

The normal picture of fibrinogen polymerization involves the removal of two fibrinopeptides (i.e., fibrinopeptides A and B) from fibrinogen, which is normally rich in α-helices, leading to its self-association through “knobs and holes,” but with otherwise no major changes in secondary structure. 77 80

Coagulopathies occur when the rate of clot formation or dissolution is unusually fast or slow, and in the case of chronic inflammatory diseases, these seem largely to coexist as hypercoagulation and hypofibrinolysis, arguably implying a common cause. 74 In a series of papers, we have shown in several diseases, such as stroke, 94 95 96 type 2 diabetes, 93 97 Alzheimer's disease, 79 98 99 and hereditary hemochromatosis, 92 that the fibrin clots induced by added thrombin adopted the form of “dense matted deposits” instead of their usual “spaghetti-like” appearance. The same kinds of effect could also be induced by unliganded (i.e., free) iron, 92 100 101 102 although no molecular explanation was (or could be) given. We pick this up in the Amyloid-Like Conformational Transitions in Fibrin(ogen) section. First however, we need to deal with two other topics.

### Endotoxin-Induced “Disseminated Intravascular Coagulation”

Endotoxin (LPS) may also induce a runaway form of hypercoagulation 57 103 104 105 106 107 108 109 110 111 112 113 114 115 116 117 known as disseminated intravascular coagulation (DIC). There is significant evidence now that DIC is reasonably well defined 46 118 119 120 and that it can directly lead to MOF and death (Cunningham and Nelson, 121 and see the following). We hypothesize here that the form of clotting in DIC in fact involves autocatalytic fibrin(ogen) self-organization leading to amyloid formation, which is consistent with the faster clot formation in the presence of endotoxin 98 and which we have recently shown can occur in vitro with miniscule amounts of LPS. 80 In particular, this may be a major contributor to the various stages of sepsis, SIRS, MODS, and ultimately of organismal death.

### Prions, Protein Free Energies, and Amyloid Proteins

Although it was originally shown that at least some proteins, when denatured and renatured, could revert to their original conformation, 122 123 implying that this was (isoenergetic with) the one of lowest free energy, this is now known not to be universal. Leaving aside chaperones and the like, one field in which proteins of the same sequence are well known to adopt radically different conformations, with a much more extensive β-sheet component (that is indeed thermodynamically more stable), is that of prion biology. 124 125 Thus, the prion protein is normally in an α-helix-rich conformation known as PrP ^c^ . However, it can also adopt a proteinase K-resistant form of the same sequence, known as PrP ^Sc^ . 126 127 128 129 130 131 The PrP ^c^ and PrP ^sc^ conformations and the catalysis of the conversion to itself by the latter of the former are very well known. The key point for us here, however, is indeed that this definitely implies 77 124 125 128 132 133 134 135 136 that proteins that may initially fold into a certain, ostensibly “native,” conformation can in fact adopt stable and more β-sheet-rich conformations of a lower free energy, separated from that of the original conformation by a potentially significant energy barrier.

### Amyloid-Like Conformational Transitions in Fibrin(ogen)

As mentioned earlier, the general view (also see the following) is that no major secondary structural changes occur during normal fibrin formation. 77 85 87 However, we know of at least three circumstances in which fibrin can (i.e., is known to) adopt a β-sheet-rich conformation: (1) in the case of specific mutant sequences of the fibrinogen a chain, 137 138 139 140 141 142 143 (2) when fibrin is stretched mechanically beyond a certain limit, 144 145 146 147 148 149 150 and (3) when formed in the presence of certain small molecules, including bacterial LPS. 76 80 151 Thus, it is well established that fibrin can form β-sheet-rich amyloids, although it is assumed that conventional blood clotting involves only a “knobs-and-holes” mechanism, without any major changes in secondary structure. 85 86 87 88 89 90 152 153 We hypothesize here that the “dense matted deposits” seen earlier are in fact β-sheet-rich amyloids, and that it is this coagulopathy in particular that contributes significantly to the procession of sepsis along or through the cascade of toxicity outlined in Fig. 1 . To be specific, we consider that the binding of the LPS must be to fibrinogen itself since only this is preexisting and we have demonstrated it directly using isothermal calorimetry. 80 We note too that there is almost no “free” LPS except immediately after its addition/liberation from a bacterium, and that the kinetics of fibrinogen polymerization during thrombin-induced clotting are so fast that it is not necessary to invoke subsequent binding of LPS to protofibrils and so on as part of the mechanism of amyloidogenesis and toxicity.

In particular, thioflavin T (ThT) is a stain whose fluorescence (when excited at 440–450 nm or so) is massively enhanced upon binding to β-sheet-rich amyloids 154 155 156 157 158 159 160 161 162 163 (whose conformation differs markedly from that of “normal” β-sheets in proteins, else it would stain most such proteins). Fig. 3A to C show micrographs taken of clots with a Zeiss superresolution microscope using Airyscan technology (Carl Zeiss), and Fig. 3D shows a micrograph taken using a Zeiss confocal microscope (see legend for specific detail). Fig. 3A is a micrograph of healthy platelet poor plasma (PPP) with added ThT and thrombin. This is a representative micrograph to show “normal” clot structure, whereas Fig. 3B and D shows healthy PPP with added LPS and ThT. High-resolution Airyscan technology ( Fig. 3B ) shows ThT binding to areas where β-sheet-rich amyloids were induced by LPS. Fig. 3C shows PPP preexposed to LPS, followed by exposure to LPS-binding protein, ThT, and thrombin. LPS-binding protein was able to reverse the formation of the β-sheet-rich amyloids areas created by preexposure to LPS. Confocal microscopy ( Fig. 3D ) also shows this ThT binding to β-sheet-rich amyloid areas. However, individual binding areas are not as clearly visible as with the Airyscan technology. Nonetheless, the extent of β-amyloid formation in the LPS-treated over the two controls is very striking.

We also note the important analyses of Strickland et al to the effect that β-amyloid can interact with fibrin(ogen) 164 165 166 167 168 169 170 and cause it to become refractory to fibrinolysis. 170 171 172 173

## Inflammatory Nature of Fibrin

The fact that fibrin itself is, or can be, inflammatory is well established 108 174 175 176 177 178 179 and does not need further elaboration. Our main point here is that in none of these studies has it been established whether (or to what extent) the fibrin is in an amyloid form or not so far. Certainly, it is very well established that amyloids can be inflammatory. 180 181 182 183

## Further Evidence for the “Trigger” Role of LPS in Large-Scale Amyloid Formation

In our previous studies, 80 we found that LPS (endotoxin) at a concentration of just 0.2 ng/L could trigger the conversion of some 10 ^8^ times more fibrinogen molecules, 80 and that the fibrin fibers so formed were amyloid in nature. (A very large amplification of structural molecular transitions could also be induced by LPS in a nematic liquid crystal. 184 185 186 ) Only some kind of autocatalytic processes can easily explain this kind of polymerization, just as occurs in prions, 77 129 131 where iron may also be involved. 70 71 187 188 189 To be explicit, the only feasible explanation is one in which an initial fibrinogen molecule with bound LPS adopts, at least on the loss of its fibrinopeptides, conformations in which the subsequent fibrinopeptide-less fibrinogens must also change their conformations to bind to it and so on as fibrinogens become protofibrils, protofibrils become fibrils, and so on. Put another way, if LPS is the only (and highly substoichiometric) addition to thrombin-induced fibrin formation, there must be an “autocatalytic process,” somewhat analogous to a prion, that must be taking place since rather than having conventional strands of fibrin, we have amorphous, denatured β sheets.

## Cytotoxicity of Amyloids

Cytotoxicity of amyloids is so well known 98 182 190 191 192 193 194 195 196 197 that it barely needs rehearsing. However, the relative toxicities of soluble material, protofibrils, fibrils, and so on are less well understood, 198 in part because they can equilibrate with each other even if added as a “pure” component (of a given narrow NB equilibrate range). Although the larger fibrils are much more easily observable microscopically, there is a great deal of evidence that it is the smaller ones that are the more cytotoxic. 199 200 201 202 203 204 205 206 207 208 209 210 211 212 213 214 215 216 So far as is known, almost all (cf. Holm et al 217 ) the established forms of amyloid are cytotoxic. However, the tests have not yet been performed for the fibrin version since it has only very recently been discovered. 77 80 This is an urgent task for the future.

## Sequelae Consistent with the Role of Amyloids in the “Sepsis Cascade” to Organ Failure and Death

If vascular or systemic amyloidogenesis really is a significant contributor to the worsening patient conditions as septic shock moves toward MOF/MODS and death, with the cytotoxic amyloids (whether from fibrin and/or otherwise) in effect being largely responsible for the MOF, then one might expect it to be visible as amyloid deposits in organs such as the kidney (whether as biopsies or postmortem). It is certainly possible to find evidence for this, 218 219 220 221 222 223 and our proposal is that such amyloid should be sought using ThT or other suitable staining in autopsy tissue.

## Hypo- or Hypertensive States

A hallmark of most of the chronic, inflammatory diseases that we have considered here and elsewhere (as cited) is that they are either normotensive or (to varying degrees) hypertensive. By contrast, sepsis and septic shock are strongly hypotensive (accompanied by hypoperfusion), 224 225 226 227 228 and their normalization is considered a crucial factor for lowering mortality. Consequently, this bears a brief discussion. Of course, at one level, it is common in biology that something can be a stimulus (e.g., of blood pressure) at a low concentration and can be an inhibitor at a high concentration (this is known as “hormesis” 229 230 231 ). At a descriptive level, this is clearly happening here. As it stands, however, we can find no literature that has compared changes in tension as the dose of endotoxin is varied, with the doses given in such studies of endotoxin-induced shock normally being sufficient to induce significant hypotension. 232 233 234 It is, however, of considerable interest that this endotoxin-induced hypotension (and other sequelae) could be relieved by antithrombin (e.g., 233 235 236 237 238 239 240 241 242 243 244 245 246 247 ), implying a contributing role for coagulopathies in the hypotension otherwise observed, albeit other mechanisms are possible. 238

## How Might This Understanding Lead to Improved Treatment Options?

Over the years, there have been many high-profile failures of therapies for various aspects of severe inflammation, sepsis, septic shock, and SIRS. These include therapies aimed at endotoxin itself (Centoxin), 248 249 250 and the use of recombinant activated protein C 251 or Drotrecogin alfa. 2 250 252 253 Anticytokine and anti-inflammatory treatments have also had, at best, mixed results. 254 255

**Fig. 4:**
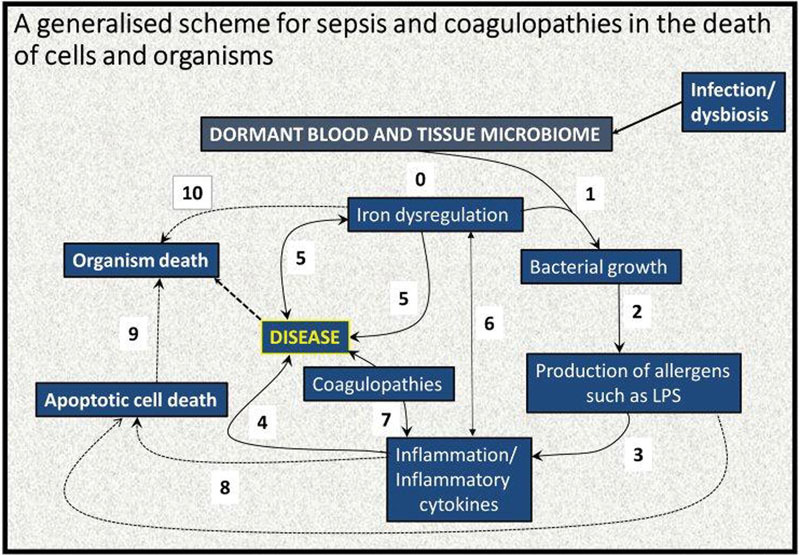
A systems biology model of the development of coagulopathies during sepsis, systemic inflammatory response syndrome, and multiple organ dysfunction syndrome. An elementary systems biology model of how iron dysregulation can stimulate dormant bacterial growth that can, in turn, lead to antigen production (e.g., of lipopolysaccharide [LPS]) that can then trigger inflammation leading to cell death 70 71 and a variety of diseases. While it is recognized that this simple diagram is very far from capturing the richness of these phenomena, there is abundant evidence for each of these steps, starting with ( **0** ) an infection/gut dysbiosis and the creation of a (dormant) blood and tissue microbiome. This is typically accompanied by ( **1** ) iron dysregulation, which is known to be present in many diseases, as both cause and result ( **5** ) and as an important cause of inflammation ( **6** ) and even organism death ( **10** ). Iron, in turn, feeds bacterial growth ( **2** ), leading to production of, for example, LPS with an accompanying upregulated inflammatory cytokine profile ( **3** ), leading to disease ( **4** ). In inflammation both apoptotic death ( **8** ) and coagulopathies ( **7** ) are well-known phenomena. In turn, apoptotic death can lead to organism death ( **9** ).

However, the overall picture that we have come to is given in Fig. 4 . This implies that we might hope to stop the progress of the sepsis/SIRS/MODS cascade at any (preferably several 256 ) of several other places, including through iron chelation, 70 71 257 258 the use of anti-inflammatory agents, the use of anticoagulants such as heparin 178 or antithrombin, 233 238 243 and the use of stimulants of fibrinolysis. 259 The success of heparin 260 261 (see also Zarychanski et al, van Roessel et al, and Okamoto et al 262 263 264 ) is especially noteworthy in the context of the present hypothesis, though it may have multiple (not simply directly anticoagulant) actions. 265 266 The same is true of antithrombin. 233 235 236 237 238 239 240 241 242 243 244 245 246 247 267 268 However, antithrombin has also not been efficacious, especially in combination with heparin 269 and is globally not recommended in sepsis therapy guidelines. 4 270 Indeed, suppressing coagulation in sepsis globally may be inimical, as it is thought to serve a protective role in the initial stages of the disease. 271 It is also noteworthy that high-density lipoprotein (HDL) cholesterol is a protective against sepsis 18 272 273 274 (HDL are antioxidant 275 and anti-inflammatory 276 and can also bind and neutralize endotoxin 277 278 279 ). Therefore, the beneficial role of certain statins in sepsis 280 281 should be seen in the context of their much more potent anti-inflammatory role 70 rather than in any (modest) role involved in lowering overall serum cholesterol. Phospholipid emulsions may also serve. 282

We have noted previously (reviewed in Kell and Pretorius 74 ) that such “dense matter deposits” (now recognized as amyloid forms) are much more resistant to fibrinolysis than is “normal” fibrinogen. The working hypothesis here is that the β-sheet-rich forms are more resistant to proteolysis because (as in prions, where the structures are known) the residues normally targeted by the relevant proteases are no longer exposed. Clearly, the removal of such structures would benefit from the development of novel proteases to which they are susceptible.

Recombinant soluble human thrombomodulin (TM-α) is a novel anticoagulant drug and has been found to have significant efficacy in the treatment of sepsis-based DIC, 247 283 284 285 286 287 288 289 290 291 292 293 albeit fully powered randomized trials are awaited, 294 295 again adding further weight to our hypothesis. As Okamoto et al 264 point out, “In the European Union and the USA, the 2012 guidelines of the Surviving Sepsis Campaign do not recommend treatment for septic DIC. 4 296 In contrast, in Japan, aggressive treatment of septic DIC is encouraged,” 297 298 299 300 and that “that Japan is one of the countries that most effectively treats patients with septic DIC.” 264 A recent meta-analysis of randomized controlled trials for the efficacy and safety of anticoagulant therapy demonstrated that such therapy has a survival benefit in those with sepsis-induced DIC, but not in the overall population with sepsis or even in populations with sepsis-induced coagulopathy. 271 We note that the influence of soluble TM may be mediated by its indirect thrombin inhibition by binding and not localizing it to a site where protein C is activated.

Thus, if it is accepted that the type of fibrin that is formed is substantially of the amyloid variety, then anticoagulant and other drugs that inhibit or reverse such amyloid processes should also be of value, 301 as they seem to be in Alzheimer-type dementia. 165 302 303

## Concluding Remarks

**Fig. 5:**
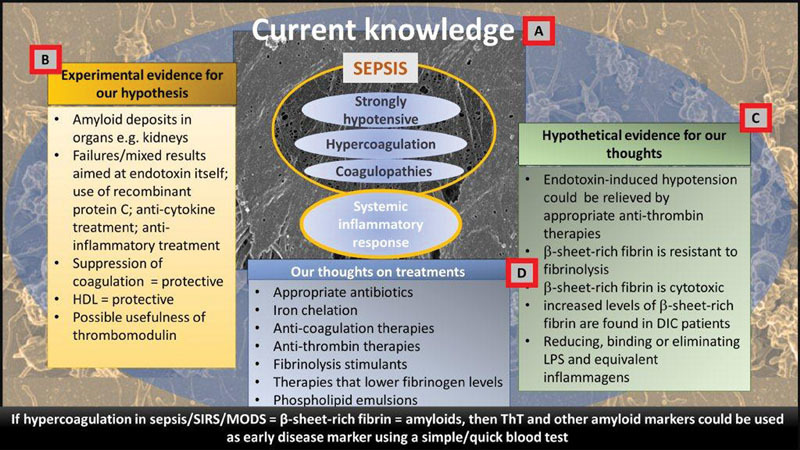
A schematic representation outlining our hypothesis based on current knowledge **(A)** , experimental evidence for our hypothesis **(B)** , hypothetical evidence ( **C** ), and finally our thoughts on (new) treatment regime approaches and early disease diagnosis ( **D** ).

We conclude by showing our line of thought in Fig. 5 . There is by now abundant evidence that coagulopathies involving fibrin clots are a major part of sepsis, SIRS, septic shock, MODS, DIC, and organismal death. We have invoked further evidence that the type of fibrin involved is an amyloid form and have suggested that it is this that is especially damaging. This definitely needs to be tested further, for instance, using appropriate stains 155 304 and/or X-ray measurements 305 306 307 in concert with cellular toxicity assays. The former could easily be performed in or near the intensive therapy unit. LPS and other substances have now been shown to cause anomalous forms of fibrin, which opens up many novels lines of work, such that reducing or eliminating them might be worthwhile, for example, with LPS-binding protein. Finally, as a corollary of the above, we suggest that anticoagulant therapies that inhibit or reverse those β-amyloid forms of fibrin production will be especially valuable. To this end, lowering the levels of fibrinogen itself would seem to be a desirable aim. 308
